# Undergraduate point-of-care ultrasound education in Japan: a nationwide cross-sectional survey of curriculum status, barriers, and implications for educators

**DOI:** 10.1186/s12909-026-09180-0

**Published:** 2026-04-10

**Authors:** Toru Yamada, Masanaga Yamawaki, Takahiro Shinohara, Hiroyuki Ichige, Suguru Mabuchi, Takuma Kimura, Takeshi Ishida, Masayoshi Hashimoto

**Affiliations:** 1 Department of General Medicine, Graduate School of Medical and Dental Sciences, Institute of Science Tokyo, 1-5-45 Yushima, Bunkyo-Ku, Tokyo, 113-8510 Japan; 2 Department of Medical Education Research and Development, Graduate School of Medical and Dental Sciences, Institute of Science Tokyo, Bunkyo-Ku, Tokyo, Japan; 3 Department of Community Medicine (Ibaraki), Graduate School of Medical and Dental Sciences, Institute of Science Tokyo, Bunkyo-Ku, Tokyo, Japan; 4 Department of R&D Innovation for Home Care Medicine, Graduate School of Medical and Dental Sciences, Institute of Science Tokyo, Bunkyo-Ku, Tokyo, Japan

**Keywords:** Point-of-care Ultrasound, Undergraduate medical education, Curriculum implementation

## Abstract

**Background:**

Point-of-care ultrasonography (POCUS) is increasingly recognized as an essential bedside clinical skill and a valuable educational tool in undergraduate medical education. Although many medical schools worldwide have begun integrating POCUS into their curricula, the extent to which undergraduate POCUS education has been formally implemented and accredited in Japan remains unclear. Therefore, this study aimed to investigate the accreditation status and implementation of undergraduate POCUS curricula across Japanese medical schools.

**Methods:**

We conducted a nationwide, cross-sectional, web-based survey of all 82 Japanese medical schools between July and September 2025. The questionnaire addressed institutional characteristics, perceived educational need for POCUS, accreditation status of undergraduate POCUS curricula, curriculum structure and content, instructional methods, assessment strategies, and perceived barriers and facilitators to implementation. Descriptive statistics were used to summarize findings, and reported barriers to implementation were compared between institutions with and without accredited curricula.

**Results:**

Sixty valid responses were analyzed (response rate: 73.2%). Although 81.6% of respondents agreed that POCUS should be integrated into undergraduate medical education, only 12 institutions (20.0%) had a formally accredited POCUS curriculum. Among accredited programs, 58.3% implemented longitudinal curricula spanning multiple academic years, most commonly integrating POCUS into clinical clerkships and physical examination teaching. All programs included diagnostic POCUS, with abdominal applications being the most frequently taught, whereas procedural POCUS was included in fewer than half of programs. Post-training assessments were conducted in 58.3% of institutions, primarily through practical skill evaluations. Major barriers to implementation included shortages of trained faculty (75.0%), limited equipment and educational resources (66.7%), lack of institutional leadership to drive curriculum development (58.3%), and insufficient curricular time. No statistically significant differences in perceived barriers were observed between institutions with and without accredited curricula (χ^2^ test, all *p* values > 0.05).

**Conclusions:**

Despite broad recognition of the educational value of POCUS, formal accreditation and educational infrastructure remain limited in Japanese medical schools. Institutions that have adopted POCUS commonly employ longitudinal and clinically integrated approaches. These findings highlight a substantial gap between perceived educational need and practical implementation and provide actionable insights for educators and curriculum leaders to prioritize faculty development, resource allocation, and institutional support for undergraduate POCUS education.

**Supplementary Information:**

The online version contains supplementary material available at 10.1186/s12909-026-09180-0.

## Background

Point-of-care ultrasonography (POCUS) is a technique in which a treating clinician independently performs ultrasonographic imaging at the patient’s bedside, acquires and interprets the images, and immediately integrates the findings into clinical decision-making [1]. The term POCUS refers to focused ultrasonographic examinations performed at the bedside by the treating clinician and is not limited to a specific organ system or procedural technique. The key characteristics of this approach include focused assessment targets and simplified, standardized evaluation criteria, allowing clinicians across various specialties and training levels to perform ultrasound with consistent and reliable quality. In addition, POCUS has been shown to enhance patient safety and support the delivery of rapid, cost-effective care [1, 2]. Historically, ultrasonography was primarily performed by specialists in radiology and cardiology; however, in recent decades its use has expanded and is now widely incorporated into postgraduate education for emergency physicians and intensivists and increasingly integrated into training for internal medicine physicians, residents, and even undergraduate medical education [1–3].

In addition to the noninvasive nature of ultrasonographic examination, the characteristics of POCUS—namely, focused assessment targets and standardized evaluation criteria that ensure consistent quality—make it highly compatible with undergraduate medical education. The real-time visual feedback provided by ultrasound devices serves as an effective tool for active learning, and studies have shown that it enhances medical students’ understanding and motivation to learn [4–6]. Considering its usefulness as an adjunct to physical examination, the introduction of ultrasound education early in medical training represents a potentially valuable approach [3, 7]. Furthermore, POCUS enables students to directly visualize anatomical structures and physiological processes in real time, thereby facilitating the integration of basic sciences with clinical reasoning. POCUS has also been reported to improve diagnostic accuracy and clinical decision-making when integrated into bedside assessment, and several studies have suggested that its use may contribute to improved patient care through more accurate and timely clinical evaluation [6, 8–11]. For these reasons, introducing POCUS during undergraduate medical education may help students develop a more structured approach to clinical reasoning and patient evaluation, while preparing them for the increasing use of ultrasound in modern clinical practice [6, 8–11]. In the United States, the adoption of POCUS curricula has progressed significantly [2, 3, 12], and undergraduate POCUS training has expanded rapidly over the past decade [2, 13]. A national survey conducted in 2020 reported that 57% of medical schools had an accredited POCUS curriculum in place [2]. However, reported barriers include a lack of trained faculty, limited curricular time within existing programs, and insufficient educational equipment [2].

In Japan, students enter medical school directly after high school and become eligible for the national medical licensing examination after completing a six-year undergraduate medical program. The “Model Core Curriculum for Medical Education” established by the Ministry of Education, Culture, Sports, Science, and Technology explicitly highlights the importance of understanding ultrasonographic examination [14]. Against this educational background, there is a substantial need for ultrasound education for medical students in Japan. Although some medical schools have reported efforts to incorporate POCUS into undergraduate education [6], comprehensive information regarding its implementation status and educational content across Japan remains limited, and no nationwide survey on this topic has been conducted in recent years.

Therefore, in the current study, we conducted a web-based survey targeting all 82 medical schools in Japan to investigate accreditation status, educational content, and barriers to the dissemination of POCUS curricula in undergraduate medical education.

## Methods

### Study design

We conducted a cross-sectional study targeting all medical schools in Japan authorized to confer a Doctor of Medicine (MD) degree. The survey was conducted between July and September 2025. The study received approval from the Institutional Review Board at the Institute of Science, Tokyo.

### Survey design

This study was conducted as a web-based questionnaire survey. All 82 Japanese medical schools (42 national medical schools, eight public medical schools, 31 private medical schools, and one defense medical school) were included. Survey invitations were distributed through two routes: postal invitations sent to the dean of each medical school and email invitations sent through a national mailing list comprising faculty members responsible for undergraduate medical education at medical schools nationwide. First, a formal invitation letter was mailed to the dean of each medical school, directing the recipient to complete the survey via a URL. In parallel, an email invitation was sent through the mailing list to educational leaders involved in undergraduate medical education at medical schools nationwide.

The questionnaire was developed with reference to a previously conducted national survey of POCUS curricula in the United States [2], with modifications to reflect the Japanese context. It consisted of 36 items covering institutional characteristics, perceived need for POCUS education, presence or absence of a formally accredited curriculum, details of educational content where applicable, and perceived barriers and facilitators to POCUS implementation (questionnaire: Supplementary Item 1). In this study, a formally accredited POCUS curriculum was defined as a POCUS educational program that had been officially approved as part of the undergraduate medical curriculum by the medical school’s curriculum committee or an equivalent institutional authority. A pilot test was conducted to assess the clarity and interpretability of the questionnaire items and to estimate completion time. The average completion time was approximately 5 min.

To ensure that one response per medical school was included in the analysis, the questionnaire included items regarding the respondent’s institution, job title, and survey invitation route (postal invitation to the dean or mailing list). Duplicate responses were identified using these items, and in cases of duplication, responses submitted via the dean-directed postal invitation were prioritized.

### Data analysis

Descriptive data analysis was performed using frequency and percentage distributions. A chi-square test was used to assess the presence of barriers between institutions with and without POCUS curriculum implementation. All analyses were conducted using STATA software (version 17.0; StataCorp LLC, College Station, TX, USA).

## Results

### Survey responses and institutional characteristics

A total of 64 survey responses were collected, including 25 received via the mailing list and 39 via postal invitations. After excluding four duplicate submissions from the same institution, 60 responses were included in the analysis (response rate: 73.2%). All participating institutions consented to the study. The most common geographic location of the responding medical schools was the Kanto region, followed by the Chubu and Kyushu–Okinawa regions. The average number of students per class was 114, and 44 (73.3%) of medical schools had more than one campus (Table 1).

**Table 1:** Characteristics of medical schools (*n* = 60/82; response rate: 73.2%)

Characteristics	Total	*n* = 60
Location	*n*	(%)
Hokkaido	1	(1.7)
Tohoku	6	(10.0)
Kanto	20	(33.3)
Chubu	11	(18.3)
Kansai	9	(15.0)
Chugoku	4	(6.7)
Kyushu–Okinawa	10	(16.7)

Number of students per academic year	Mean	(SD)
	114	1.62
Number of campuses	***n***	(%)
1	16	(26.7)
2	19	(31.7)
3	14	(23.3)
4	4	(6.7)
5 or more	7	(11.7)
Status of POCUS curriculum accreditation		
Accredited	12	(20.0)
Not accredited but scheduled	0	(0.0)
Not accredited (no plan for accreditation)	48	(80.0)
POCUS should be incorporated into the curriculum		
Strongly agree	20	(33.3)
Agree	29	(48.3)
Neutral	10	(16.7)
Disagree	1	(1.7)
Strongly disagree	0	(0.0)

*POCUS* Point-of-care ultrasound

### Current status of accredited POCUS curricula

Among the 60 responding institutions, only 12 (20.0%) had a formally accredited POCUS curriculum. The remaining 48 schools reported that their curricula were not accredited, and none had specific plans for accreditation in the following academic year. However, 49 schools (81.6%) agreed that POCUS should be integrated into undergraduate curricula (Strongly agree: 33.3%; Agree: 48.3%) (Table 1).

### Implementation status and structure of the POCUS curriculum

Table 2 shows the details of POCUS implementation among the 12 institutions delivering an accredited curriculum. POCUS curricula were implemented across all academic years, with the highest implementation rate in the fifth year (91.7%), followed by the fourth and sixth years (75.0% for both). Three institutions (25.0%) introduced POCUS, starting in the first year. Longitudinal curricula spanning multiple academic years were found in seven programs (58.3%). Interdisciplinary curricula were also observed, with POCUS most frequently integrated into clinical clerkships (100.0%), followed by physical examination (58.3%), and preclinical subjects, such as anatomy, physiology, and pathology.

**Table 2:** Implementation year and longitudinal/interdisciplinary integration in POCUS curricula (*n* = 12)

Implementation Year*	Institutions	(%)	Mandatory	(%)	Elective	(%)	Mandatory & Elective	(%)
First year	3	(25.0)	1	(8.3)	2	(16.7)	0	(0.0)
Second year	5	(41.7)	3	(25.0)	2	(16.7)	0	(0.0)
Third year	4	(33.3)	2	(16.7)	1	(8.3)	1	(8.3)
Fourth year	9	(75.0)	5	(41.7)	2	(16.7)	2	(16.7)
Fifth year	11	(91.7)	2	(16.7)	4	(33.3)	5	(41.7)
Sixth year	9	(75.0)	1	(8.3)	5	(41.7)	3	(25.0)
Longitudinal curriculum	7	(58.3)						
Interdisciplinary curriculum*								
Anatomy	2	(16.7)						
Physiology	2	(16.7)						
Pathology	1	(8.3)						
Physical assessment	7	(58.3)						
Clinical clerkship	12	(100.0)						

*POCUS* Point-of-care ultrasound

*Multiple answers allowed

Clinical physicians served as the primary instructors for all POCUS programs (100.0%) (Table 3). By specialty, Gastroenterology and Hepatobiliary Medicine was the most commonly involved department (58.3%), followed by General Medicine (50.0%), and Emergency Medicine and Cardiology (both 41.7%) (Supplementary Item 2). In some institutions, residents and medical students also participated in the instruction. Ultrasound devices used for education ranged from smartphone-sized portable devices to large cart-based machines. All institutions used both lectures and hands-on sessions. Lectures were primarily delivered in person (83.3%), with only two using online formats. Hands-on instruction was mostly conducted in person, although one program incorporated live remote instruction. Seven programs (58.3%) conducted post-education assessments. Image acquisition skills were evaluated mainly through practical assessments, such as objective structured clinical examinations, while image interpretation was assessed primarily through written examinations. The most common educational goal was enabling students to use POCUS as a clinical decision-making tool (83.3%), with only a minority incorporating procedural guidance as a learning objective (16.7%).

**Table 3:** Overview of POCUS education: Instructors, devices, methods, and competencies (*n* = 12)

Characteristics	***n***	**(%)**
Professions involved in POCUS education*		
Clinical physician	12	(100.0)
Basic science physician	3	(25.0)
Senior resident	1	(8.3)
Junior resident	1	(8.3)
Medical student	1	(8.3)
Sonographer	1	(8.3)
Ultrasound devices used for education*		
Portable ultrasound (smartphone-sized)	5	(41.7)
Portable ultrasound (tablet-sized)	3	(25.0)
Notebook or small cart type	8	(66.7)
Large cart type	7	(58.3)
POCUS teaching methods*		
Lecture		
In person	10	(83.3)
Online	2	(16.7)
Hands-on		
In-person instruction (simulator)	11	(91.7)
In-person instruction (live model)	12	(100.0)
Live online instruction (simulator)	1	(8.3)
Self-directed learning (simulator)	6	(50.0)
Post-training evaluation*		
Image acquisition skill evaluation	7	(58.3)
Practical assessment (e.g., OSCE/SDOT)	6	(50.0)
Review of ultrasound images	3	(25.0)
Image interpretation skill evaluation	7	(58.3)
Practical assessment (e.g., OSCE/SDOT)	3	(25.0)
Review of ultrasound images	2	(16.7)
Written examination	6	(50.0)
Required competencies*		
Can understand the basic principles and indications of US	9	(75.0)
Can understand the limitations of POCUS	6	(50.0)
Can use ultrasound as a tool for understanding anatomy and physiology	3	(25.0)
Can use ultrasound as a tool to support bedside clinical decision-making	10	(83.3)
Can use ultrasound as a tool for diagnosis and exclusion of disease	6	(50.0)
Can use ultrasound as an aid for invasive procedures	2	(16.7)

*POCUS* Point-of-care ultrasound, *OSCE* Objective structured clinical examination, *SDOT* Standardized direct observation tool

*Multiple answers allowed

**Fig. 1:**
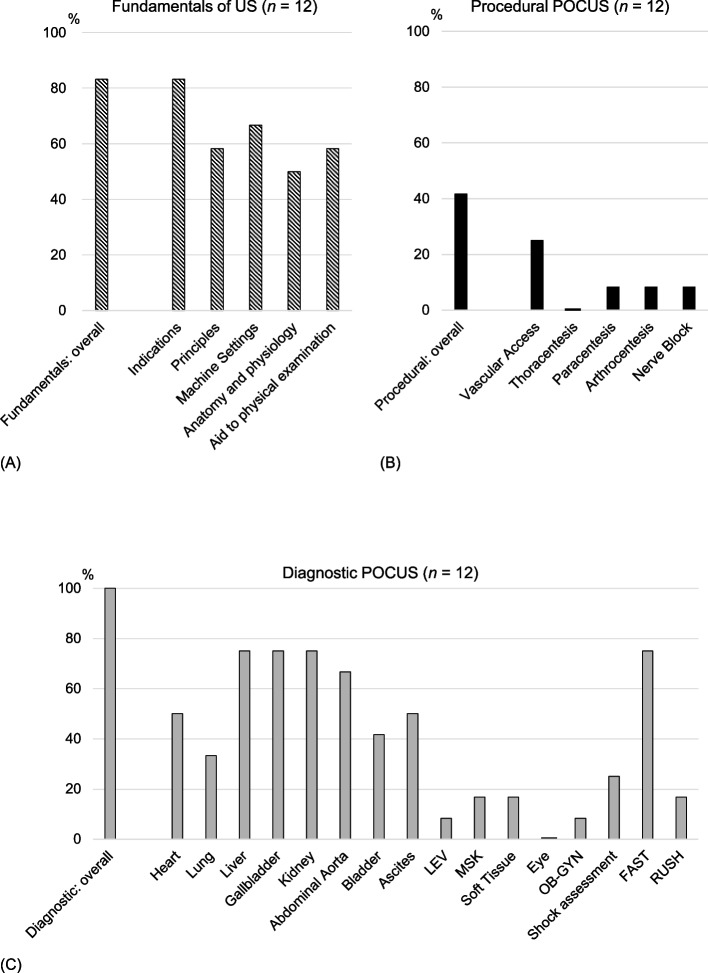
Educational content covered in each category: **A** fundamentals of ultrasound, **B** procedural POCUS, and **C** diagnostic POCUS—based on responses from 12 medical schools with an accredited POCUS curriculum (multiple selections allowed)

Regarding educational content, 10 programs (83.3%) taught the fundamentals of ultrasound, and all programs included diagnostic POCUS. Procedural POCUS was taught in five programs (41.7%). Detailed content by organ system and application type is shown in Fig. 1A–C. Among diagnostic POCUS applications, abdominal examinations were the most commonly taught.

### Barriers to POCUS curriculum implementation

**Fig. 2:**
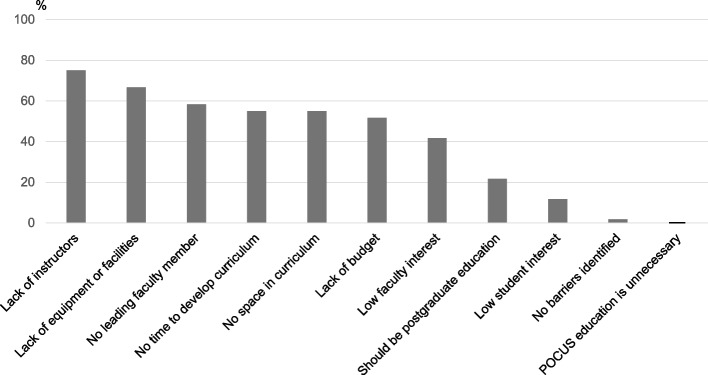
Barriers to implementing a POCUS curriculum were collected from all 60 respondent medical schools, regardless of accreditation status

Figure 2 shows the barriers to implementing a POCUS curriculum, as reported by all 60 institutions. The most frequently cited barriers were a lack of trained instructors (75.0%), followed by insufficient equipment and resources (66.7%), and lack of leadership to drive curriculum implementation (58.3%). Additional barriers reported by more than half of the institutions included insufficient time for curriculum development (55.0%), lack of space in the existing curriculum (55.0%), and insufficient budget (51.7%). Notably, the proportion reporting low interest in POCUS was higher among instructors than among students (instructor interest: 41.7%; student interest: 11.7%). Only one institution reported no barriers. There were no statistically significant differences in reported barriers between institutions with and without an accredited POCUS curriculum (χ^2^ test, all *p* values > 0.05) (Supplementary Item 3).

## Discussion

This study represents the first nationwide survey of undergraduate POCUS curricula among all 82 medical schools in Japan. The high response rate of 73.2% suggests that the findings provide a realistic reflection of the current state of undergraduate POCUS education in Japan. Only 20% of medical schools had a formally accredited POCUS curriculum in place. In contrast, a majority of institutions (81.6%) agreed that POCUS should be integrated into undergraduate education, indicating a clear gap between educational needs and curricular formalization. Among accredited programs, approximately 60% offered a longitudinal curriculum, and all institutions reported integrating POCUS into interdisciplinary curricula, particularly within clinical clerkships. The most frequently reported barriers to implementation were a lack of qualified instructors (75.0%), insufficient equipment and resources (66.7%), and an absence of leadership to drive implementation (58.3%). These findings demonstrate that, while the importance of POCUS education is widely recognized across Japanese medical schools, standardization and formal curricular adoption remain limited, highlighting a substantial gap between recognition and implementation.

Compared with the United States, where more than half of medical schools have implemented formally approved POCUS curricula [2], the implementation rate in Japan remains substantially lower. This contrast highlights a gap between the perceived need for POCUS education and the formal establishment of curricula within Japanese undergraduate medical education. In the current survey, the implementation rate of accredited POCUS curricula at Japanese medical schools was only 20%, with no institutions planning future accreditation. Although differences in medical education systems must be considered, the implementation of POCUS in Japan appears to be lagging behind international trends. Notably, however, more than 80% of the institutions in the current study agreed that POCUS should be integrated into the curriculum, and the inclusion of ultrasonographic understanding in the national Model Core Curriculum suggests that the institutional and conceptual framework is already in place to support broader adoption [14]. This finding suggests that the stagnation of undergraduate POCUS education in Japan is unlikely to be caused by a lack of awareness of POCUS, but rather by delays in practical curriculum development and faculty training at the institutional level.

In this study, the implementation of POCUS curricula spanned all academic years, from the first year through to the sixth year. These findings suggest that POCUS can function as a longitudinal educational tool integrated throughout undergraduate medical training. A previous survey in the United States reported that approximately 8% of institutions had longitudinal POCUS curricula [2]. However, this comparison should be interpreted cautiously because of differences in sample size and potential variation in the definition of longitudinal curricula between studies. Although the overall implementation rate remains low, the findings indicate that, among institutions that have adopted POCUS, a certain degree of longitudinal and integrated education centered around POCUS has been achieved. The high rate of integration with physical examination and clinical clerkships suggests that POCUS is being positioned not merely as an imaging modality but as a bedside diagnostic imaging tool integrated into clinical assessment [15–17]. However, education on procedural POCUS was limited, with only 16.7% of institutions identifying the use of POCUS as an adjunct to invasive procedures as a learning objective. Given the level of procedural proficiency expected at the undergraduate level and considerations for patient safety, the current emphasis on using POCUS as a bedside decision-support tool appears to be appropriate.

The most frequently cited barrier to implementing a POCUS curriculum was a lack of qualified instructors (75.0%). This challenge has also been reported in other countries [2, 3, 12] and is not unique to Japan. All 12 accredited institutions reported that clinical physicians were responsible for POCUS instruction, whereas only three institutions had involvement from basic science physicians. Because clinical physicians typically balance both clinical and teaching responsibilities—and few possess substantial experience with POCUS—the development of a robust instructional framework is challenging. We found that although student interest in POCUS was high, faculty interest was comparatively low (percentage reporting low interest in POCUS: students 11.7% vs. faculty 41.7%), suggesting that such human resource constraints may contribute to this disparity. Furthermore, postgraduate surveys in Japan have repeatedly identified the shortage of POCUS instructors as a primary barrier [18]. This suggests that the issue is not merely a lack of technical skills but reflects a broader structural challenge: the absence of an adequate system for faculty development to train educators who can teach POCUS effectively.

Barriers such as insufficient equipment and resources, limited curricular space, and inadequate budgets were also reported by more than half of the institutions, in accord with findings from studies in other countries [2, 3, 12]. Ultrasound equipment generates revenue in clinical settings, but it does not directly produce financial returns when used for educational purposes. Therefore, relying solely on educational budgets to secure an adequate number of devices poses inherent limitations. Because ultrasound education is explicitly highlighted in the Model Core Curriculum for Medical Education, developed by the Ministry of Education, Culture, Sports, Science and Technology in Japan [14], establishing a comprehensive support system through collaboration among governmental bodies, academic societies, and industry is essential.

This study represents the first national survey of all medical schools in Japan on POCUS curricula, providing a quantitative overview of the current implementation status and associated challenges. The data obtained can serve as foundational evidence for discussions and policy development aimed at standardizing POCUS education, fostering faculty development, and advancing curriculum implementation in Japan.

### Limitations

The current study has several limitations that should be considered. First, the study was based on a web-based survey, with all data relying on self-reported responses from representatives at each institution. Therefore, respondents’ perceptions may not fully reflect actual operational practices. Additionally, faculty members who were more interested in POCUS may have been more likely to respond, introducing a potential selection bias.

Second, as a cross-sectional study, this research does not capture temporal changes or the process of POCUS curriculum adoption in Japan. Given that POCUS education is undergoing rapid transformation because of technological advancement and increasing device availability, future studies incorporating multiple time points are warranted.

Third, the survey focused on the presence, structure, and barriers of the POCUS curricula, and did not directly evaluate educational quality or learning outcomes (such as learner competence, objective structured clinical examination performance, or clinical skill improvement). Consequently, we were unable to draw conclusions about the impact of POCUS education on learner performance or clinical outcomes in the current study.

## Conclusion

This nationwide survey revealed that although many medical schools in Japan recognize the educational value of POCUS, formally accredited undergraduate curricula remain limited, highlighting a gap between perceived importance and systematic implementation.

Beyond describing existing practice, this study offers actionable insights for educators seeking to bridge this gap. In particular, strengthening faculty development, establishing structured and longitudinal curricula that progress from foundational concepts such as anatomy to clinical diagnostic applications, and standardizing educational resources across institutions may support the sustainable integration of POCUS into undergraduate medical education.

Future studies should also explore the implementation of POCUS education in other healthcare professions, such as nursing and allied health programs, to better understand the broader interprofessional educational context of ultrasound training.

## Supplementary Information

Supplementary Material 1.

## Data Availability

The datasets used and/or analyzed during the current study are available from the corresponding author upon reasonable request.

## Abbreviations

OSCE: Objective structured clinical examination
POCUS: Point-of-care ultrasonography
SDOT: Standardized direct observation tool
US: Ultrasound
