# Supplementary material for: Undergraduate point-of-care ultrasound education in Japan: a nationwide cross-sectional survey of curriculum status, barriers, and implications for educators
Source: BMC Med Educ. 2026 Apr 10;26:841. doi: 10.1186/s12909-026-09180-0 (PMC13217953; doi:10.1186/s12909-026-09180-0)
Supplement: Supplementary file 1 — Supplementary Material 1. [file 12909_2026_9180_MOESM1_ESM.docx]

Supplementary Material

**Undergraduate point-of-care ultrasound education in Japan: A nationwide cross-sectional survey of curriculum status, barriers, and implications for educators**

Toru Yamada^a^, Masanaga Yamawaki^b^, Takahiro Shinohara^a^, Hiroyuki Ichige^c^, Suguru Mabuchi^a^, Takuma Kimura^d^, Takeshi Ishida^c^, Masayoshi Hashimoto^a^

^a^Department of General Medicine, Graduate School of Medical and Dental Sciences, Institute of Science Tokyo, Bunkyo-ku, Tokyo, Japan

^b^Department of Medical Education Research and Development, Graduate School of Medical and Dental Sciences, Institute of Science Tokyo, Bunkyo-ku, Tokyo, Japan

^c^Department of Community Medicine (Ibaraki), Graduate School of Medical and Dental Sciences, Institute of Science Tokyo, Bunkyo-ku, Tokyo, Japan

^d^Department of R&D Innovation for Home Care Medicine, Graduate School of Medical and Dental Sciences, Institute of Science Tokyo, Bunkyo-ku, Tokyo, Japan

**CONTACT** Toru Yamada, MD, PhD, Department of General Medicine, Graduate School of Medical and Dental Sciences, Institute of Science Tokyo, 1-5-45 Yushima, Bunkyo-ku, Tokyo 113-8510, Japan. Email: toru.y.fmed@tmd.ac.jp. Tel: +81-3-5803-5229. Fax: +81-3-5803-0276.

**Supplementary Item 1**

**Web-based questionnaire items**

1. Please select the name of your university (in alphabetical order in Japanese).

2. Through which route are you submitting this response?

□ From official request letters sent to each university

□ From the medical education mailing list

3. Please indicate your department/affiliation and position.

4. Do you agree that the aggregated, anonymized results of this survey may be published in academic papers or conference presentations?

□ Yes

□ No

5. How many students are enrolled per academic year in your school of medicine?

6. How many campuses does your university have?

□ 1

□ 2

□ 3

□ 4

□ 5 or more

7. Do you think POCUS education should be incorporated into undergraduate medical education?

□ Strongly agree

□ Agree

□ Neutral

□ Disagree

□ Strongly disagree

8. At your medical school, is any form of POCUS education formally accredited as part of the university curriculum (regardless of organ system or content)?

□ Yes →Q 9

□ No – scheduled for future accreditation →Q 9

□ No – no plan for accreditation →Q 36 (Barriers)

**Details of** **POCUS Education**

9. Is POCUS education conducted at a specific facility such as a simulation center?

□ Yes

□ No

10. Which of the following best describes your POCUS curriculum?

□ Mandatory

□ Elective

□ Both (partly mandatory and partly elective)

11. What types of POCUS education are provided? (Multiple answers allowed)

□ Fundamentals of ultrasound

□ Diagnostic POCUS (e.g., focused cardiac, lung, abdominal examination)

□ Procedural POCUS (e.g., US-guided puncture)

12. What are the specific contents of the “fundamentals of ultrasound”? (Multiple answers allowed)

□ Indications for ultrasound examination

□ Principles of ultrasound

□ Basic machine settings

□ Ultrasound for understanding basic anatomy and/or physiology

□ Ultrasound as an aid to physical examination

□ Not implemented

□ Other (specify)

13. What are the contents of “diagnostic POCUS”? (Multiple answers allowed)

□ Heart □ Lung □ Liver □ Gallbladder □ Kidney □ Abdominal aorta □ Bladder

□ Ascites □ Lower extremity vessels □ Musculoskeletal □ Soft tissue

□ Obstetrics / Gynecology □ Eye □ Shock assessment □ FAST □ RUSH

□ Not implemented □ Other

14. What are the contents of “procedural POCUS”? (multiple answers allowed)

□ Vascular access (e.g., central venous catheter guidance)

□ Thoracentesis □ Paracentesis □ Arthrocentesis □ Nerve block

□ Not implemented □ Other

15. Is longitudinal POCUS education conducted across multiple years?

□ Yes

□ No

16–21. Is POCUS education included in the curriculum of each academic year?

(Select Mandatory / Elective / Both / Not applicable for each of Years 1–6.)

22. Is POCUS education integrated into the teaching of anatomy?

□ Yes

□ No

23. Is POCUS education integrated into the teaching of physiology?

□ Yes

□ No

24. Is POCUS education integrated into the teaching of pathology?

□ Yes

□ No

25. Is POCUS education included in the teaching of physical examination?

□ Yes

□ No

26. Is POCUS education incorporated into clinical clerkship?

□ Yes

□ No

27. Is POCUS education provided as a stand-alone course on ultrasound examination?

□ Yes

□ No

28. What teaching methods are used? (Multiple answers allowed)

□ Lecture – in-person □ Lecture – online

□ Hands-on (in-person using simulator)

□ Hands-on (in-person peer-to-peer)

□ Hands-on (in-person with standardized patient model)

□ Hands-on (in-person with real patients)

□ Hands-on (remote live instruction with simulator)

□ Hands-on (remote live instruction peer-to-peer)

□ Hands-on (remote live instruction with standardized patient)

□ Hands-on (remote live instruction with real patients)

□ Self-practice in skills lab □ Other

29. Who are the instructors responsible for POCUS education? (Multiple answers allowed)

□ Clinical faculty □ Basic science faculty □ Senior residents □ Junior residents

□ Medical students □ Sonographers □ Other

30. Which clinical departments are involved in POCUS education? (Multiple answers allowed)

Cardiology / Pulmonology / Gastroenterology and Hepatology / Endocrinology-Metabolism / Nephrology / Hematology / Neurology / Rheumatology / General Medicine / Critical Care / Emergency Medicine / Obstetrics & Gynecology / Pediatrics / Radiology / Anesthesiology / Surgery (Gastrointestinal / Hepatobiliary-Pancreatic) / Thoracic Surgery / Cardiovascular Surgery / Orthopedic Surgery / Urology / Other

31. Which devices are used for POCUS education? (Multiple answers allowed)

□ Portable ultrasound (hand-held, smartphone size)

□ Portable ultrasound (tablet size)

□ Notebook / Small cart-type

□ Large cart-type

32. Is any assessment of learning effect performed after POCUS education?

□ Yes

□ No

33. How are knowledge and image-interpretation skills assessed? (Multiple answers allowed)

□ Practical assessment (e.g., OSCE / SDOT)

□ Review of ultrasound images submitted by students

□ Written test

□ Other

□ Not assessed

34. How are image-acquisition skills assessed? (Multiple answers allowed)

□ Practical assessment (e.g., OSCE / SDOT)

□ Review of images submitted by students

□ Other

□ Not assessed

35. What level of POCUS understanding is expected upon completion of the curriculum? (Multiple answers allowed)

□ Can understand the basic principles and indications of ultrasound

□ Can understand the limitations of POCUS

□ Can use ultrasound as a tool for understanding anatomy and physiology

□ Can use ultrasound as a tool to support bedside clinical decision-making

□ Can use ultrasound as a tool for diagnosis and exclusion of disease

□ Can use ultrasound as an aid for invasive procedures

□ Other

**Barriers to Implementing a POCUS Curriculum** **(Multiple answers allowed)**

36. Which of the following are barriers to implementing a POCUS curriculum at your medical school?

□ Lack of qualified instructors

□ Lack of ultrasound equipment or facilities

□ Insufficient time to develop the curriculum

□ No space in the existing curriculum to add POCUS education

□ Lack of budget for POCUS education

□ Belief that POCUS should be taught after graduation (internship or residency)

□ Low student interest

□ Low faculty interest

□ Lack of leadership to spearhead POCUS education

□ POCUS education is unnecessary

□ No barriers

□ Other (specify)

**Supplementary Item 2**

| Departments involved in POCUS education | | |
| --- | --- | --- |
| **Departments** | ***n*** | *%* |
| Cardiology | 5 | (41.7) |
| Pulmonology | 0 | (0.0) |
| Gastroenterology and Hepatobiliary | 7 | (58.3) |
| Endocrinology and Metabolism | 3 | (25.0) |
| Nephrology | 1 | (8.3) |
| Hematology | 0 | (0.0) |
| Neurology | 0 | (0.0) |
| Rheumatology | 1 | (8.3) |
| General Medicine | 6 | (50.0) |
| Intensive Care Medicine | 3 | (25.0) |
| Emergency Medicine | 5 | (41.7) |
| Obstetrics and Gynecology | 2 | (16.7) |
| Pediatrics | 0 | (0.0) |
| Radiology | 4 | (33.3) |
| Anesthesiology | 2 | (16.7) |
| Gastrointestinal and Hepatobiliary Surgery | 1 | (8.3) |
| Thoracic Surgery | 0 | (0.0) |
| Cardiovascular Surgery | 0 | (0.0) |
| Orthopedic Surgery | 1 | (8.3) |
| Urology | 1 | (8.3) |
| Other | 2 | (16.7) |
| POCUS: point-of-care ultrasound | | |

**Supplementary Item 3**

| Comparison of barriers to POCUS curriculum implementation between accredited and nonaccredited universities | | | | | | |
| --- | --- | --- | --- | --- | --- | --- |
| **Barriers** | **Total (*n*)** | **%** | **Accredited (*n*)** | **Not Accredited (*n)*** |  | ***p*** |
| Lack of instructors | 45 | 75 | 11 | 34 |  | 0.136 |
| Lack of equipment or facilities | 40 | 66.7 | 9 | 31 |  | 0.494 |
| No leading faculty member | 35 | 58.3 | 7 | 28 |  | 1.000 |
| No time to develop curriculum | 33 | 55.0 | 8 | 25 |  | 0.364 |
| No space in curriculum | 33 | 55.0 | 8 | 25 |  | 0.364 |
| Lack of budget | 31 | 51.7 | 7 | 24 |  | 0.605 |
| Low faculty interest | 25 | 41.7 | 5 | 20 |  | 1.000 |
| Should be postgraduate education | 13 | 21.7 | 3 | 10 |  | 0.754 |
| Low student interest | 7 | 11.7 | 0 | 7 |  | 0.159 |
| No barriers identified | 1 | 1.7 | 0 | 1 |  | 0.614 |
| POCUS education is unnecessary | 0 | 0 | 0 | 0 |  | - |
| POCUS: point-of-care ultrasound | | | | | | |
